# Awareness of the relative quality of spatial working memory representations

**DOI:** 10.3758/s13414-022-02646-5

**Published:** 2023-01-31

**Authors:** Alison Y. Li, Thomas C. Sprague

**Affiliations:** Department of Psychological and Brain Sciences, University of California, Santa Barbara, Santa Barbara, CA USA

**Keywords:** Eye Movements, Cognitive, Memory, Visual working and short-term memory, Decision making

## Abstract

Working memory (WM) is the ability to maintain and manipulate information no longer accessible in the environment. The brain maintains WM representations over delay periods in noisy population-level activation patterns, resulting in variability in WM representations across items and trials. It is established that participants can introspect aspects of the quality of WM representations, and that they can accurately compare which of several WM representations of stimulus features like orientation or color is better on each trial. However, whether this ability to evaluate and compare the quality of multiple WM representations extends to spatial WM tasks remains unknown. Here, we employed a memory-guided saccade task to test recall errors for remembered spatial locations when participants were allowed to choose the most precise representation to report. Participants remembered either one or two spatial locations over a delay and reported one item’s location with a saccade. On trials with two spatial locations, participants reported either the spatial location of a randomly cued item, or the location of the stimulus they remembered best. We found a significant improvement in recall error and increase in response time (RT) when participants reported their best-remembered item compared with trials in which they were randomly cued. These results demonstrate that participants can accurately introspect the relative quality of neural WM representations for spatial position, consistent with previous observations for other stimulus features, and support a model of WM coding involving noisy representations across items and trials.

## Introduction

Exploring our modern environment frequently requires making decisions based on representations of objects that may not be directly in view. For example, when driving, it is impossible to simultaneously view the traffic surrounding us in all directions—instead, we iteratively sample the environment by looking through various windows and mirrors, and maintain representations of the location(s) of the other cars around us over these brief interruptions. Sometimes, we are sure we know the position of a recently viewed car, while other times, we feel the need to double-check our surroundings. This highlights the question: How do we know how “good” our representations of remembered locations are?

Such scenarios require the use of working memory (WM), which enables the brief maintenance of limited amounts of information no longer directly in view, and is thought to place fundamental constraints on cognition (Cowan, 2001; Luck & Vogel, 2013; Ma et al., 2014). WM is supported by the sustained activity of neural population codes distributed throughout the brain, including many regions of the cerebral cortex (Bays, 2015; Christophel et al., 2017; Curtis & Sprague, 2021; Serences, 2016; Sreenivasan & D’Esposito, 2019). Because neural population activity is noisy, WM representations encoded within these activity patterns fluctuate across items and trials. Indeed, models involving random fluctuations in the representation of each remembered item outperform those lacking such variability (van den Berg et al., 2012; van den Berg et al., 2014). Moreover, participants appear to be sensitive to these random fluctuations: Behavioral measures of recall precision track behavioral measures of uncertainty and/or confidence (Geurts et al., 2022; Honig et al., 2020; Li et al., 2021; Rademaker et al., 2012; van den Berg et al., 2017; Vandenbroucke et al., 2014; Yoo et al., 2018), and the quality of information represented by neural activation patterns in visual and parietal cortex correlates with behavioral recall performance and confidence reports on individual trials (Geurts et al., 2022; H.-H. Li et al., 2021). Altogether, these results point to a model whereby WM representations of individual objects are instantiated within noisy neural activity patterns, and participants “read out” both the represented information and the uncertainty with which that information is represented from these neural activity patterns when making WM-guided decisions. Returning to the driver’s seat of the car: This model suggests that you are aware of the quality of the neural representation of the remembered spatial location and can use this understanding to guide your next decision.

Beyond accurately introspecting the quality of a single remembered item, or one of several remembered items randomly cued by the experimenter, participants are additionally able to infer the relative quality of each of several WM representations. When participants are allowed to report all items in a display in an order of their own choosing (Adam et al., 2017; Adam & Vogel, 2017), or asked to report the item they believe they can recall most accurately (Fougnie et al., 2012; Suchow et al., 2017; Williams et al., 2022), behavioral performance is better for the first-reported item (Adam et al., 2017) or the best-remembered item (Fougnie et al., 2012; Suchow et al., 2017; Williams et al., 2022). These studies offer convincing evidence that participants can monitor and use the relative quality of multiple WM representations when guiding decisions. However, these studies focused on representations of stimulus features such as color, orientation, or shape, and have used stimulus arrays in which each remembered item is presented at a different location. When objects occupy disjoint locations, this may allow for independent neural codes (i.e., nonoverlapping pools of neurons) to represent the feature(s) of each independent item, and thus, for the “quality” of each item to be assayed based on the relative quality of each independent neural code (Geurts et al., 2022; H.-H. Li et al., 2021). Instead, when multiple spatial positions must be maintained, if spatially selective (i.e., retinotopic) neural populations encode those positions, it is then necessarily the case that multiple feature values (positions) are encoded in the same population as distinct hills or bumps of activity. In such a scenario, an alternative readout strategy which incorporates the simultaneous representations of multiple items would be necessary. Thus, it remains unknown whether participants can similarly introspect the relative quality of multiple WM representations for spatial position. Based on previous findings that participants can accurately report the quality of a single remembered spatial WM representation (H.-H. Li et al., 2021), or one of several spatial WM representations that is randomly cued (Yoo et al., 2018), and that participants can accurately decide which of several feature WM representations is best-remembered (Adam et al., 2017; Fougnie et al., 2012; Suchow et al., 2017; Williams et al., 2022), we predicted that participants would similarly be able to decide which of several remembered spatial locations they could report most accurately. Instead, if it is impossible to accurately compare the relative quality of multiple spatial representations encoded within the same pool of neurons, then we would expect participants cannot improve their performance when allowed to decide which location to report. Additionally, the previous studies establishing that participants can compare the quality of multiple WM representations have been unable to measure RTs, which offer a useful opportunity to establish differences in decision processes between WM task conditions (Pearson et al., 2014; Schneegans & Bays, 2016, 2018). If participants must directly compare their judgments of each WM representation’s quality before deciding which to report, we would expect longer RTs when participants make a decision about which item to report as compared with trials in which they are randomly cued. Instead, if participants are able to immediately report the stronger of the two representations without a direct comparison process, RTs would be faster when the best item could be reported.

Here, we tested whether participants can correctly intuit the best of several spatial WM representations on a trial-by-trial basis by employing a memory-guided saccade task which allowed us to precisely characterize both the accuracy of memory reports and response time on each trial. Participants remembered the location of either one or two objects on each trial, and reported one location with a saccadic eye movement after a delay period. Critically, on trials requiring participants to remember two items, participants either reported one item that was randomly cued, or they reported the item they believed they could report most accurately. To summarize our results, when participants could report their best item, recall error was lower and response times were slower than on trials in which they reported a cued item. Further analyses suggested it was unlikely that participants were adopting a strategy in which they reported locations that they could, on average, recall more precisely, suggesting that their judgments of the best-remembered item were based on trial-by-trial fluctuations in the quality of each WM representation. These results support a model in which WM representations of multiple spatial positions fluctuate trial by trial, and demonstrate that participants can read out and compare the quality of each representation when making WM-guided decisions.

## Methods

### Participants

We recruited 21 healthy adult human participants, including one of the authors (*n* = 20; female = 17; *M*_*age*_ = 23.33 years, *SD*_*age*_ = 2.63), to participate in the experiment for either monetary compensation or course credit ($10/hr or 1 credit/hr). The Institutional Review Board at University of California Santa Barbara approved all human subjects procedures. Prior to participation in the study, all participants provided written informed consent. All participants reported normal or corrected-to-normal vision and were 18 years of age or older. The data from one participant were removed from analysis because they could not complete the experiment due to technical difficulty acquiring eye tracking data, so the final dataset includes *n* = 20 participants. The sample size was determined based on previous reports using similar methods (e.g., continuous report tasks; Fougnie et al., 2012, *n* = 10, 700 trials per condition; Suchow et al., 2017, *n* = 12, 200 trials per condition), which reported an effect size of *d*_z_ = 0.84 and 0.55, respectively, for a similar comparison (paired *t* test between a randomly cued and best-remembered condition). We aimed to detect an effect size of at least *d*_z_ = 0.70 with 80% power, which required a sample size of *n* = 19, which we rounded up to *n* = 20.

### Design and procedures

**Fig. 1:**
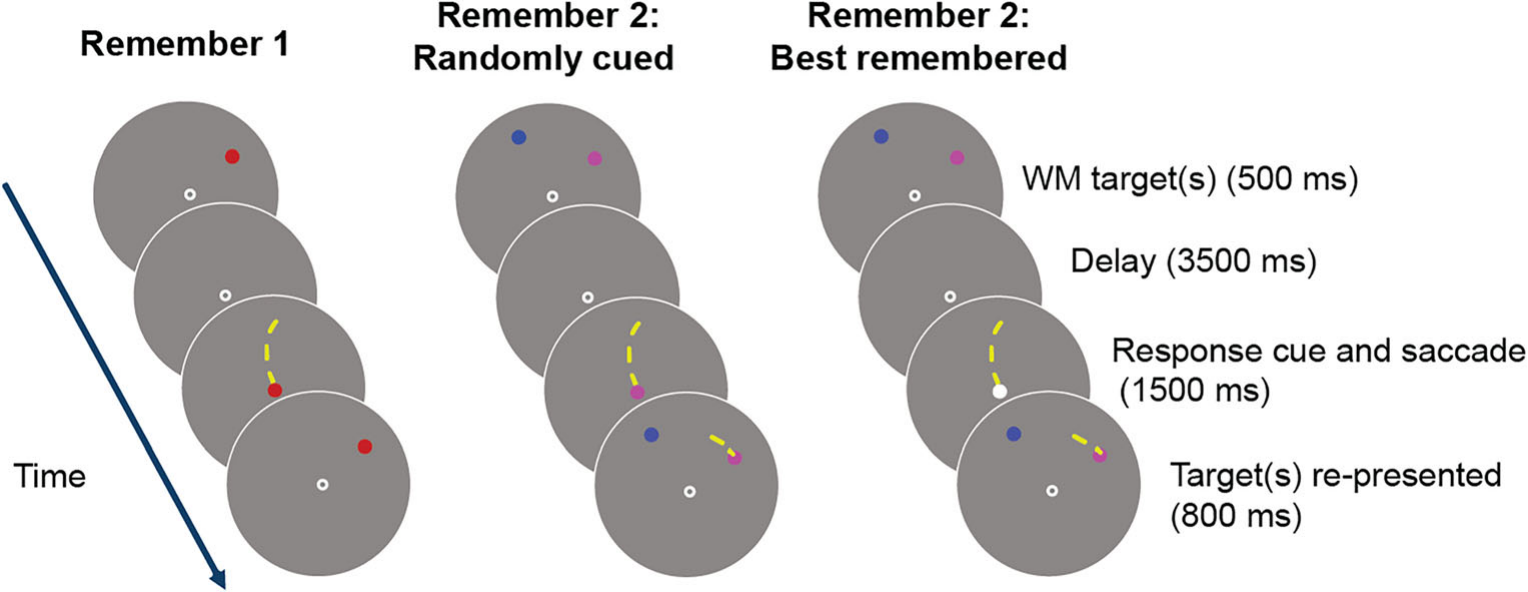
Task design. Participants were instructed to remember the location of one or two colored dots presented at random location(s) on an invisible ring 12° from fixation while maintaining fixation at a fixation point at the center of a large circular aperture. After a 3.5-s delay, participants made a memory-guided saccade towards a remembered location in one of three conditions. On “Remember 1” (R1) trials, the fixation response cue always matched the color of the remembered dot, and participants reported that location. On “Remember 2–randomly cued” (R2-random) trials, the fixation response cue matched one of the two dot colors, and participants reported the location of the dot with the matching color. On “Remember 2–best remembered” (R2-best) trials, the fixation response cue became white, and participants reported the location of the target they felt they could recall most precisely. After 1.5 s, the target location(s) were re-presented, and participants fixated the reported target dot before returning to fixation​. Cartoon schematic shown (not drawn to scale); see Methods for specifics of stimulus display parameters. Dashed yellow line indicates a cartoon depiction of gaze trajectory (not shown to participants). All stimuli presented within a gray circular aperture. (Color figure online)

This study investigated whether participants are aware of the relative quality of their spatial WM representations using a memory-guided saccade task (Funahashi et al., 1989; H.-H. Li et al., 2021). The study tested three conditions (Fig. 1). In the Remember 1 (R1) condition, participants remembered a single position over a delay period and reported its location with a saccadic eye movement. The other two conditions both contained two targets, each presented in a different color. In the Remember 2–randomly cued (R2-random) condition, the participant was asked to recall the location of one of the targets, randomly chosen on each trial. For the Remember 2–best remembered (R2-best) condition, the participant was asked to recall the location of one of the targets that they thought they remembered the best.

Each ~5-min run contained 30 trials, with 10 trials per condition, randomly interleaved. Following a previous report, we aimed to acquire ~200 trials per condition (Suchow et al., 2017), which required a minimum of 20 behavioral runs (range: 20 to 34, mean 22.5) split over multiple 1-hr behavioral testing sessions. All participants were exposed to all conditions of interest (repeated-measures design).

On each trial, either one or two targets, each a small dot presented in a different color, appeared in a random location on the screen within a large gray circular aperture for 500 ms (Fig. 1). Participants remembered the location of all target dot(s) presented over a subsequent 3.5-s delay period (similar to Funahashi et al., 1989), Then, a cue appeared at the center of the screen at the fixation point, instructing the participant to report the location of one of the remembered stimuli. On trials with two remembered positions (66% of trials), the cue either matched the color of one of the two targets, indicating the participant must report the location of the cued target (R2-random condition), or, it was white, indicating the participant should report the location they felt they remembered most precisely (R2-best condition). For example, if a trial had two targets (e.g., blue and purple), the cue would be one of the two colors, or it would be white. To report the remembered location of the cued target, participants made a saccade to the remembered location. Participants had 1.5 s to make their response. Then, target dots were re-presented at the remembered location(s) for 800 ms. During these 800 ms, participants fixated the location of the reported target dot. After the dots were removed from view, participants returned their gaze to the central fixation point and waited for the next trial to begin after a randomly chosen intertrial interval (2 to 4 s). We provided no behavioral feedback at the end of each run.

### Stimuli and apparatus

Participants performed the task in a dark room with an experimenter seated behind them actively monitoring their eye movements using the eye-tracking control computer throughout the session. We presented stimuli on a 25-in. Dell S2417DG LCD display (2,560 × 1,440 resolution; 30-cm height; 120-Hz refresh rate), which participants viewed from a chinrest located 56 cm away. All experimental equipment was mounted on an adjustable-height table which was raised/lowered for each participant to minimize head and neck strain.

For all sessions, we used an EyeLink 1000 Plus infrared eye tracker (SR Research) placed beneath the computer screen. The camera always tracked the participant’s right eye at 1000 Hz. We calibrated with a 13-point calibration routine at the beginning of each run. Throughout the experiment, an experimenter monitored gaze data and adjusted pupil/corneal reflection detection parameters as necessary.

The stimuli were colorful dots (0.65° radius) presented on an invisible ring that had a 12° radius from a central fixation mark. Each dot was randomly assigned a location on each trial, and the color of the dot was randomly chosen from red (RGB: 200, 0, 0; 14.49 cd/m^2^), yellow (RGB: 130, 130, 0; 31.79 cd/m^2^), blue (RGB: 0, 0, 255; 7.22 cd/m^2^), and purple (RGB: 180, 0, 180; 15.70 cd/m^2^). The locations were constrained so that in the two targets conditions the dots were always separated by at least 30° polar angle. Throughout all the trials, a gray fixation point (radius 0.3°) was presented in the center of the screen, which was filled by a circular aperture (15° dva radius) on a black background to minimize any biases induced by the edges of the rectangular stimulus display monitor. We presented stimuli and communicated with the eye tracker using MATLAB and the Psychophysics Toolbox (Version 3.0.15; Pelli, 1997).

### Gaze data processing

The main dependent variables in the current study were recall error derived from response error (i.e., the Euclidean distance between target location and endpoint of the final saccade) and response time (i.e., the time between response cue onset to the beginning of initial saccade). We extracted these variables using automated eye-tracking data preprocessing and processing routines conducted offline after the experimental session similar to those reported previously (Hallenbeck et al., 2021; H.-H. Li et al., 2021; github.com/tommysprague/iEye_ts). These procedures include removing blinks, adjusting drift over each run, recalibrating raw gaze data trial by trial, identifying memory-guided saccades, and automatically excluding trials with poor data or participant noncompliance. We implemented fully automated procedures blind to trial conditions to minimize experimenter bias. We qualitatively evaluated the efficacy of the below-described automated pre/processing steps blind to experimental conditions of each trial before conducting our primary analyses.

Specifically, blinks were defined as samples within 200 ms before and after pupil size fell below the 1.5th percentile of the distribution across pupil size samples from a run. Velocity was computed based on smoothed gaze time courses (5-ms standard deviation Gaussian kernel). Saccades were defined based on a velocity threshold of 30°/s, duration threshold of 0.0075 s, and amplitude threshold of 0.25°. Periods between saccades were defined as fixations. Drift correction for each trial was based on the modal fixation position from the trial period before the go cue. For recalibrating raw gaze data on each trial, we used the closest fixation to the target position during the 800 ms period after the target was re-presented. Then, we fit a third-order polynomial for each coordinate (*x, y*) to match the actual WM position of the nearest target and the measured gaze coordinate. Finally, we used this polynomial to recalibrate the gaze traces across trials in each run. Only trials with a fixation within 2.5° of a target stimulus were used for fitting the polynomial.

After preprocessing and automatically identifying saccades/fixations, we then automatically labeled the “initial” and “final” saccades (and their resulting endpoints) for each trial. The initial saccade was defined as the first large saccadic eye movement towards the target position (>5° amplitude, <150-ms saccade duration). The final saccade was defined as the last saccade before the target stimuli reappeared at the end of the trial. If the participant made no corrective saccade(s) after the initial saccade during a trial, the initial saccade and final saccade would be identical. We defined RT based on the onset of the initial saccade following the response cue and final saccade error as the Euclidean distance between the final saccade endpoint and the reported target. On R1 trials and R2-random trials, we computed the distance to the cued target location. On R2-best trials, we computed the distance to the target nearest to the location participants fixated when the targets were re-presented.

We implemented blinded and automated trial exclusion criteria to ensure results reflect trials which could be confidently quantified using our automated procedures. We excluded trials based on these criteria: (1) fixation failure (the participant broke fixation beyond a 2.5° radius window around fixation during the target presentation or delay epochs), (2) initial saccade with RT faster than 100 ms or slower than 1 s, or (3) no initial saccade was detected (no saccade >5° amplitude) or the initial saccade was erroneous (endpoint >5° from any target). This automated exclusion procedure resulted in excluding between 1.5 and 55% of trials per participant (mean ± *SEM*: 17 ± 3.04%). For five participants (sub002, sub006, sub009, sub014, and sub021), more than 20% of trials were excluded. When we repeated all analyses excluding these participants, our results remained the same. Importantly, the proportion of included trials did not reliably differ across the critical R2-random and R2-best task conditions (paired *t* test of proportion of trials included per subject), *t*(19) = −0.468, *p* = .645, *d*_z_ = −0.105. Interestingly, when directly comparing each R2 condition to the R1 condition, there were more trials included for the R2 conditions: R1 vs. R2-random, *t*(19) = −2.85, *p* = .01, *d*_z_ = −0.637; R1 vs. R2-best, *t*(19) = −3.22, *p* = .005, *d*_z_ = −0.720, despite the greater difficulty associated with larger set size. Altogether, this suggests that our primary results cannot be well-explained by different numbers of included trials between the conditions.

### Data analysis and statistical procedures

For the primary analyses of RT and recall error, we computed the average initial saccade RT and average Euclidean response error across all included trials within each condition per participant, then subjected these values to inferential statistical tests (one-way repeated-measures analysis of variance [ANOVA] with follow-up pairwise *t* tests as necessary; Fig. 3). For the primary analysis comparing final saccade recall error across conditions (Fig. 3a), we additionally computed a measure of precision based on the variability of the saccade endpoint distribution tangential to the target location. We aligned the final saccade endpoint across all trials within each condition by circularly rotating the endpoint around fixation such that the reported target was located at (12°, 0°). Then, we computed the standard deviation of the *y* coordinate of these aligned response endpoints for each condition and participant and subjected these values to the same inferential tests described above.

To evaluate each of several heuristic strategies, we computed correlations between two variables (see Results; Fig. 4) for each participant, then performed a one-sample *t* test on the sample of Fisher *r*-to-*z* transformed correlation values across participants against the null hypothesis of a zero correlation (two-tailed).

## Results

In the current study, participants performed a memory-guided saccade task which required them to precisely remember the location of one or two colored dots on each trial (Fig. 1). On two thirds of the trials, participants were instructed to report one remembered location (on R1 trials, there was only one location that could be cued; on R2-random trials, the fixation point changed color to match one of the dots; participants reported the matching dot’s location). On the remaining one third of the trials (R2-best trials), the fixation point turned white during the response period, which instructed participants to choose the dot location that they believed they remembered the best to report.

**Fig. 2:**
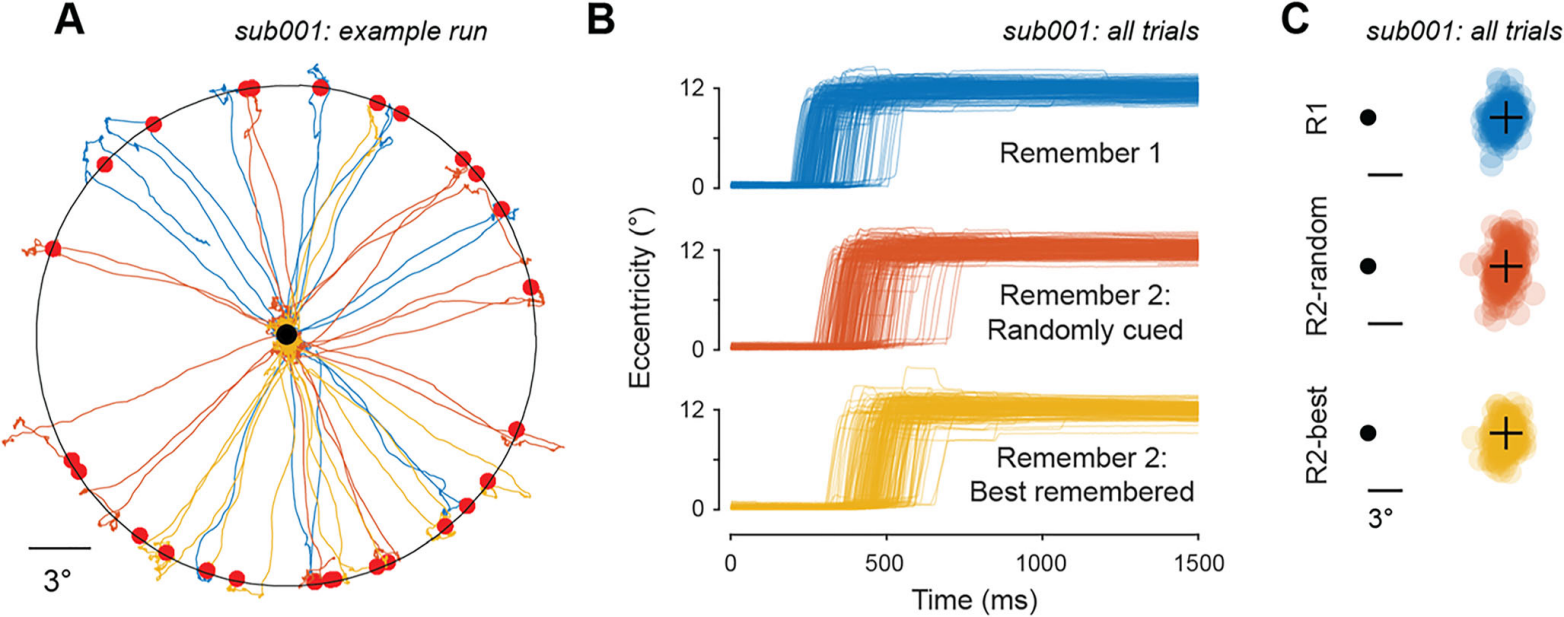
Behavioral results from an example participant. **a** Eye position traces following response cue for all trials from an example run (30 trials) from an example participant. Line color denotes condition (see **b**). Red dots indicate remembered target locations. Black ring is drawn at 12° eccentricity, and was not presented during the experiment. **b** Time course of gaze position (eccentricity) for all trials for an example participant, sorted by condition. On all trials which pass objective and blinded trial inclusion criteria, a rapid and ballistic saccade occurs shortly after the response cue. Qualitatively, saccades occur earliest for R1 trials, latest for R2-best trials, and at intermediate latencies for R2-random trials. **c** Saccade endpoints across all trials for an example participant, sorted by condition and aligned to remembered target location (black +). Endpoints are rotated around fixation to align the reported target location to the right of fixation (relative fixation location indicated by black circle). Behavioral reports are most precise on R1 trials and least precise on R2-random trials (consistent with the commonly-observed set size effect). Recall error is qualitatively improved on R2-best trials compared with R2-random trials

Qualitatively, participants accurately reported the remembered location with quick and ballistic saccadic eye movements (Fig. 2a). Comparing all trials from an example participant across the three conditions, saccades towards the target were large in amplitude (Fig. 2b), which was typical across our sample of participants. Moreover, examining these saccade amplitude traces, it is apparent that the onset of the initial ballistic saccadic eye movement after the response cue was fastest when a single item was remembered, slower when two items were remembered and a single item was cued, and slowest when the participant was required to choose the best remembered item to report. Finally, comparing the spatial distribution of saccadic endpoints between conditions for this example participant (Fig. 2c) suggests that they can most precisely report the location of a single item (R1), especially when compared with the R2-random condition. Interestingly, when they are allowed to choose which of two locations to report, the spatial recall error is qualitatively smaller (lower spatial spread of data points) than when one of two locations is randomly cued (Fig. 2c). Data from this example participant illustrate the effectiveness of our automated scoring and trial exclusion procedures (see Methods: gaze data processing). All trials included in subsequent analyses exhibit the typical time course of memory-guided saccades, with a large-amplitude initial saccade towards the remembered location followed typically by one or more corrective saccades.

**Fig. 3:**
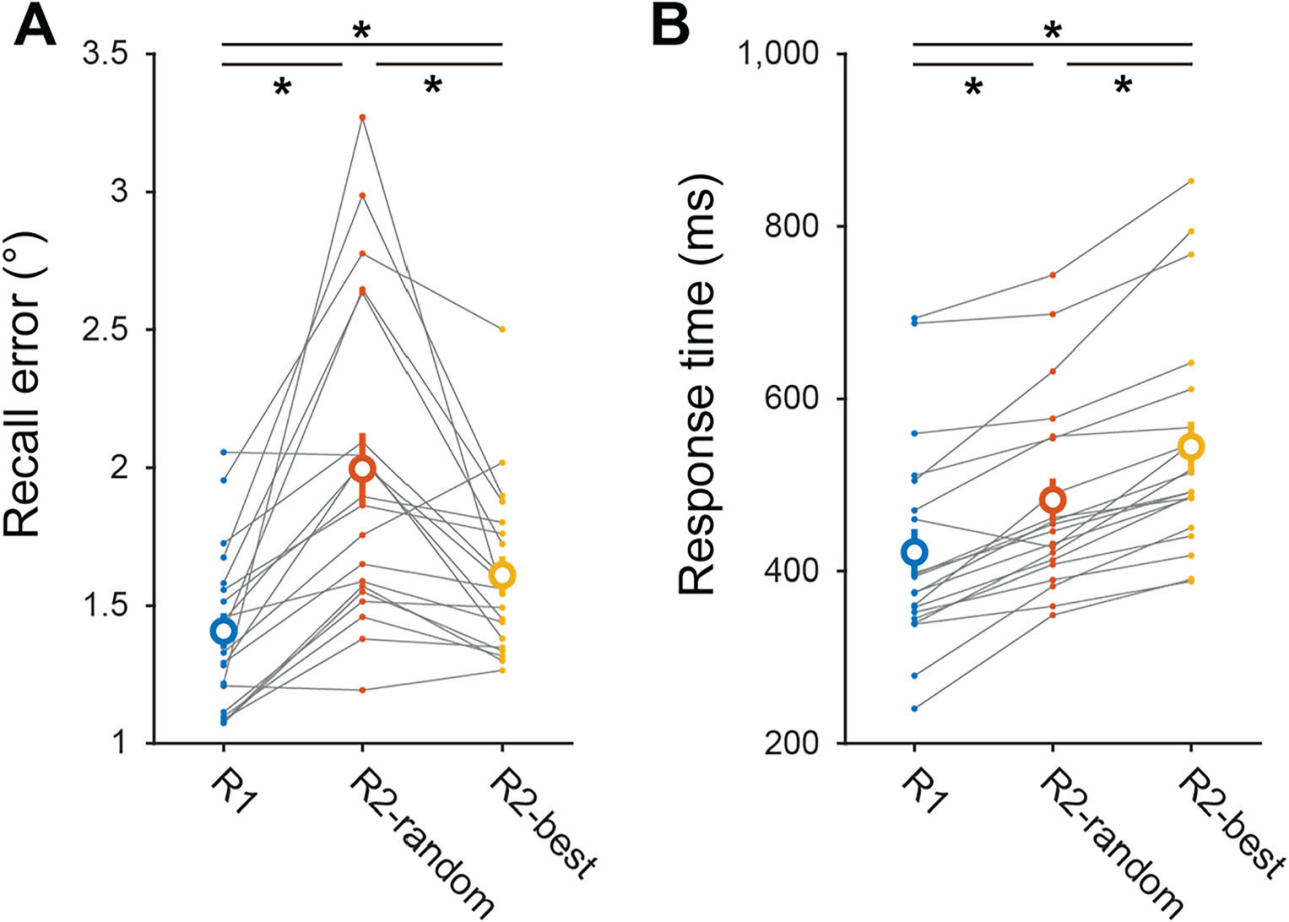
Participants are more accurate and slower to respond when reporting the best-remembered item. **a** We quantified recall error as the average Euclidean distance between the final saccade endpoint and the remembered target position (see Methods). Recall was most accurate when participants remembered a single location (R1), and dropped substantially when they remembered two locations and were randomly cued (R2-random). However, recall improved when participants reported the best-remembered item (R2-best). **b** Response times, measured based on the onset of the first saccade after the response cue (see Methods), were fastest on R1 trials and slower on R2-random trials. On R2-best trials, performance was slower still, suggesting the process of introspecting the relative quality of both WM representations takes additional time. * indicates significant difference, paired *t* test, *p* < .05 (see text). Error bars show *SEM*. (Color figure online)

To quantitatively test whether participants can introspect which of several spatial WM representations they can report most accurately, we compared WM recall error (mean Euclidean error of final saccade endpoint) and RT (onset of initial saccade) across conditions using repeated-measures one-way ANOVAs. First, we found a main effect of condition on memory-guided saccade recall error (Fig. 3a; absolute error), *F*(2, 38) = 19.605, *p* < .001, η_p_^2^ = 0.51. Follow-up paired *t* tests showed that performance was more precise in the R1 condition than both of the R2 conditions: R1 vs. R2-random, *t*(19) = −5.247, *p* < .001, *d*_*z*_ = 1.173; R1 vs. R2-best, *t*(19) = −3.281, *p* = .004, *d*_z_ = 0.734. Importantly, a direct comparison between R2-random and R2-best trials revealed a significant difference in recall error: R2-random vs. R2-best, *t*(19) = 3.685, *p* = .002; *d*_z_ = 0.824. The result was consistent with our prediction that participants can introspect the relative quality of multiple spatial WM representations, similar to previous observations using feature values like color and orientation. We also repeated these analyses using an alternative measure of precision (standard deviation of the tangential saccade endpoint distribution; see Methods), and these results were unchanged: the same pattern was found in the mean difference of recall precision between conditions. *F*(2, 38) = 29.5625, *p* < .0001; η_p_^2^ = .61. Planned contrasts revealed that all pairwise comparisons were significant: R1 vs. R2-random, *t*(19) = −7.875, *p* < .001, *d*_z_ = 1.761; R1 vs. R2-best, *t*(19) = −3.611, *p* = .002, *d*_z_ = 0.807; R2-random vs. R2-best, *t*(19) = 3.982, *p* = .001, *d*_z_ = 0.890. In addition to comparing final saccade endpoints, we also tested whether the primary saccade endpoint error (the distance between the remembered location and the location fixated after the first ballistic saccade towards the reported location) differed across task conditions. The results were qualitatively consistent with those shown in Fig. 3a, although the difference between the R2-random and R2-best conditions was not significant, *t*(19) = 2.071, *p* = .052, *d*_z_ = 0.463.

Second, we also found a main effect of condition on memory-guided saccade response time (Fig. 3b), *F*(2, 38) = 74.902, *p* < .001, η_p_^2^ = 0.80. RT was fastest in the R1 condition, slowest in the R2-best condition, and intermediate in the R2-random condition. Planned pairwise contrasts revealed that all contrasts were significant: R1 vs. R2-random, *t*(19) = −6.762, *p* < .001, *d*_z_ = 1.512; R1 vs. R2-best, *t*(19) = −9.939, *p* < .001, *d*_z_ = 2.222; R2-random vs. R2-best, *t*(19) = −7.494, *p* < .001, *d*_z_ = 1.676. The longer RT in the R2-best condition compared with the R2-random condition converges with our prediction that participants need extra time to assess the relative quality of each object’s representation before making a decision about which one to report, and rules out the possibility that the strongest WM representation can immediately be recalled more quickly on R2-best trials.

We also compared the average number of saccades made by participants in generating their response. We counted the number of saccades generated including the ballistic primary saccade towards the remembered position before the feedback cue appeared. There was no systematic deviation in the mean number of saccades across conditions (one-way repeated-measures ANOVA), *F*(2, 38) = 0.672, *p* = .516, η_p_^2^ = 0.034. This suggests that participants were not making finer-grained oculomotor adjustments on any condition compared with the others, and rules out a possible trade-off between faster RTs (time before primary saccade) and increased amount of adjustment (greater number of corrective saccades).

### Ruling out alternative explanations

These results are consistent with our prediction that participants can monitor the quality of multiple WM representations and use the relative quality of those representations to guide behavior. However, it could be the case that participants are instead implementing heuristic strategies that do not involve monitoring trial-by-trial fluctuations in each WM representation. For example, each participant may have an area of the screen that they believe they can remember more precisely. If this belief is accurate, and if participants employ a strategy whereby they report the item closest to their “best-performing” location(s) on the screen on R2-best trials, it is possible to observe results like those shown in Fig. 3a.

**Fig. 4:**
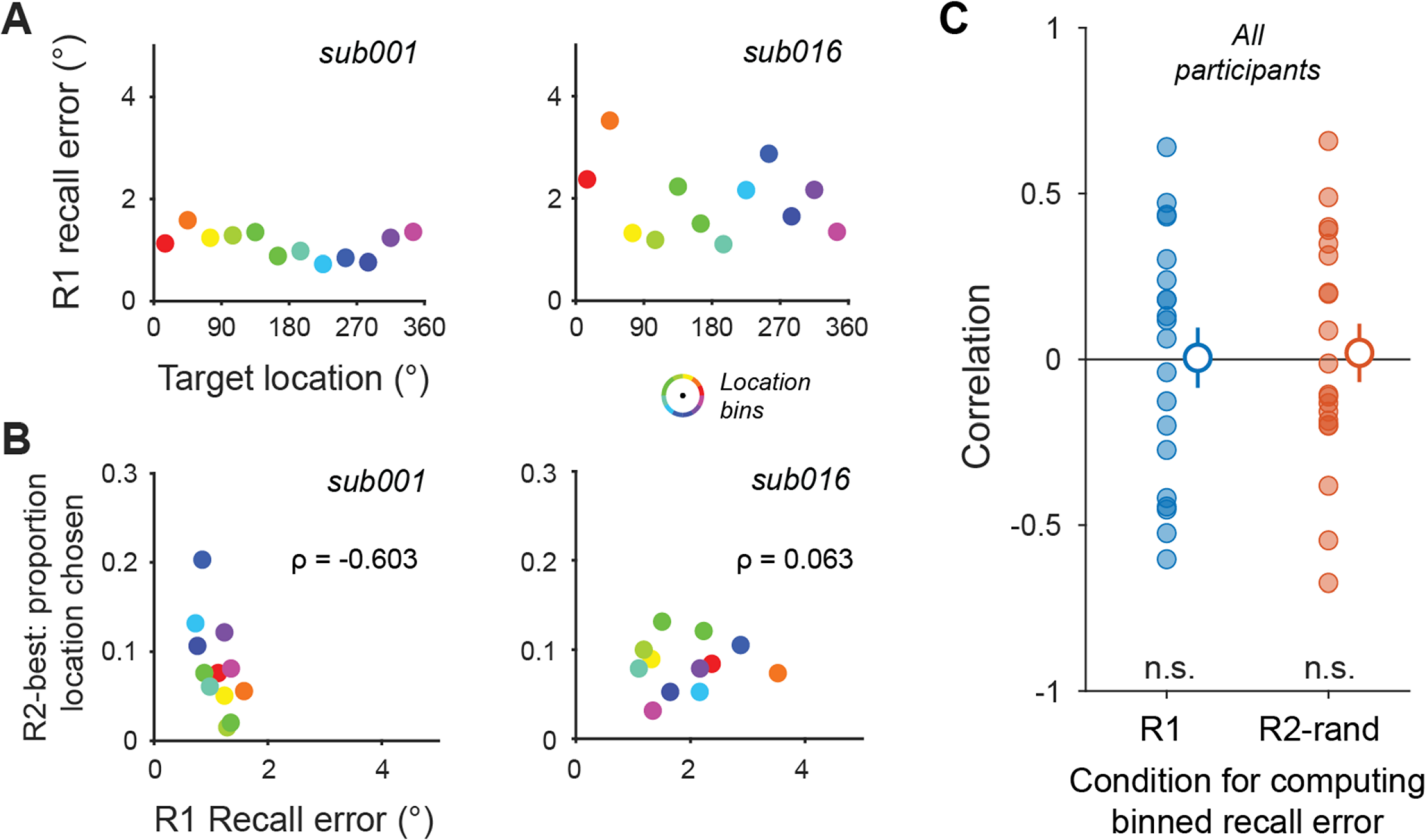
Participants did not employ a location-based heuristic on R2-best trials. We tested the possibility that participants are aware that they can report some locations better than others on average, but do not introspect the quality of their trial-by-trial WM representations by directly comparing the recall error on R1 or R2-random trials within each of 12 location bins to the likelihood that a target within that bin would be ‘chosen’ in R2-best trials. **a** For each participant (two example participants shown), we sorted trials into 12 equally spaced location bins (see inset; lower right) and computed average recall error for all trials within each bin. **b** For each participant, we plotted the likelihood that they would report a target if it was presented in each location bin on R2-best trials against the recall error on R1 trials plotted in (**a**). If all screen locations are treated equally, there should be no correlation between R1 recall error and the likelihood of reporting each location. However, if participants frequently choose targets within a location bin for which they have low recall error on R1 trials, this correlation would be negative. Correlation values (ρ) appear on each participant’s figure. **c** Correlations between R2-best: proportion location chosen and R1: Recall error (left) or R2-random: Recall error (right) across location bins (as in **b**) across all participants in our sample. While some participants do show negative correlations (e.g., sub001, panel **b**), this is not true on average across our sample. Error bars show SEM. (Color figure online)

To test this possibility, we reasoned that we could use the R1 condition to characterize which location(s) were best remembered for each participant. If, on the R2-best condition, participants default to reporting the locations they believe they can remember the best (rather than evaluating the relative quality of each representation on a trial-by-trial basis), we would expect they are more likely to choose to report the location that is better remembered, on average, on R1 trials. For each participant, we first plotted the average recall error for R1 trials sorted into 12 equally spaced position bins (Fig. 4a). For some participants (e.g., sub001), there was essentially no variation across location bins, while for other participants (e.g., sub016), there was a greater amount of variability. Then, for each location bin, we computed the proportion of R2-best trials in which participants reported the location within that bin. If, for example, a participant remembered one location bin extremely precisely compared with all others, and this knowledge alone was driving their choice of which item to report on R2-best trials, then we would expect the probability of choosing that location would be high, and the probability of choosing other locations would be lower. When we directly plotted the probability of choosing a location within a bin on R2-best trials against the average recall error for the corresponding bin on R1 trials (Fig. 4b), we observed substantial variability across participants. For some participants, this heuristic does seem to be employed (e.g., sub001), such that the participant’s “choice” is predicted by their average recall error on R1 trials. However, for other participants, this result is not observed (e.g., sub016). Note that because we use entirely separate data to compute the recall error for each location bin and to compute the likelihood of choosing an item within that bin, there is no possible circularity in the analysis procedure.

To quantify whether this heuristic is employed, on average, across our participant sample, we computed a correlation for each participant between error from R1 trials and best remembered probability across the 12 location bins. If best-remembered location bins are more likely to be chosen, we would expect to see a negative correlation on average. However, across our sample, there was no reliable difference in these correlation coefficients from zero (Fig. 4c; one-sample *t* test of Fisher *r*-to-*z* transformed correlation coefficients between R1 and best remembered probability against zero), *t*(19) = 0.05, *p* = .96, *d*_z_ = 0.012; same test based on correlation between R2-random and best remembered probability: *t*(19) = 0.22, *p* = .83, *d*_z_ = 0.05. Thus, while it may be the case that a small number of participants may, in part, adopt a location-based heuristic to report their best-remembered item, this appears to be rare, and does not account for the overall results across our sample. Finally, to ensure that a subset of participants exhibiting strong negative correlations (e.g., sub001 in Fig. 4b) did not drive the primary results shown in Fig. 3, we repeated the analyses in Fig. 3 after excluding data from the five participants with the most negative correlation in Fig. 4c (based on sorting trials using both R1 and R2-random binned recall errors) and the results remain unchanged (data not shown).

## Discussion

We investigated whether participants can directly compare the relative quality of multiple spatial WM representations to support improved behavioral performance on a memory-guided saccade task (Figs. 1 and 2). When participants remembered two locations, they reported one location more accurately when they were allowed to choose which item they could report the best compared with when they were cued to report one item’s location at random (Fig. 3a). Moreover, RTs were longest when participants were required to choose their best item to report (Fig. 3b), suggesting the involvement of a time-consuming process for comparing each item’s representation quality. Finally, we ruled out the possibility that participants only use a location-based heuristic to report their best-remembered item, because, on these trials, participants were not more likely to report the location(s) on the screen they could most accurately recall based on their performance on cued trials (Fig. 4). Altogether, these results support a model of WM whereby noisy neural population codes for multiple items encode information about the relative “quality” of each item’s representation, and this information can be read out and compared when engaging in WM-guided behavior.

Previous studies have established that increasing WM load decreases recall precision for remembered stimulus features like color (Adam et al., 2017; Adam & Vogel, 2017; Bays et al., 2009; Wilken & Ma, 2004; Zhang & Luck, 2008), orientation (Adam et al., 2017; Bays & Husain, 2008; Gorgoraptis et al., 2011), shape (A. Y. Li et al., 2022), motion direction (Emrich et al., 2013; Zokaei et al., 2011), and spatial position (Bays & Husain, 2008; Ester et al., 2018; Sprague et al., 2014, 2016; reviewed in Bays, 2015; Luck & Vogel, 2013; Ma et al., 2014). Our study using a memory-guided saccade task (Fig. 1) replicates this general finding: on trials where participants were randomly-cued to report one of two remembered locations, they responded with greater recall error than when they only remembered a single location (Fig. 3a; see also Schneegans & Bays, 2018). A key advantage of the memory-guided saccade task is that we could simultaneously achieve a continuous report of remembered location and response time on each trial. Our results replicate previous spatial recall studies measuring RTs for motor actions (finger pointing: Schneegans & Bays, 2016; memory-guided saccade: Schneegans & Bays, 2018) that manipulated set size: responses were slower when two items were remembered and one was randomly cued than when only a single item was remembered (Fig. 3b).

Our observation that participants can perform more accurately when asked to report the location of their best-remembered item when compared with a randomly cued item (Fig. 3) replicates and extends previous observations that participants can compare multiple WM representations of colored circles (Adam et al., 2017; Fougnie et al., 2012; Suchow et al., 2017; Williams et al., 2022), oriented lines (Adam et al., 2017), and cartoon cubes (Suchow et al., 2017). These previous studies are all consistent with a model of WM whereby each item is encoded with a random level of precision (e.g., “variable precision” models; van den Berg et al., 2012), and so these results support a similar model for spatial WM representations. This is an important demonstration because spatial WM tasks are commonly used in the animal physiology and computational modeling literature for probing the neural mechanisms supporting WM (Compte et al., 2000; Curtis & Sprague, 2021; Funahashi et al., 1989; Schneegans & Bays, 2016, 2018), and because deficits in spatial WM are observed in patients with schizophrenia (Cannon et al., 2005; Matthews et al., 2014; Zhao et al., 2021).

### Evaluating the quality of WM representations

Previous studies aiming to directly relate the accuracy of WM reports, confidence reports, and/or the quality of neural representations measured with neuroimaging techniques have primarily focused on tasks requiring reporting a single item. For example, Rademaker et al. (2012) required participants to remember the orientation of several gratings over a short delay interval before reproducing the remembered orientation of a randomly cued grating and giving a confidence rating. Trials with better confidence reports were those with more accurate performance on the orientation reproduction task, establishing that participants can accurately report the quality of single WM representations. Similar results are observed for a single remembered location (H.-H. Li et al., 2021), a single cued location among multiple items (Yoo et al., 2018), and a single cued color among multiple items (Honig et al., 2020). Moreover, a pair of recent fMRI studies have established a link between the quality of decoded neural representations from areas of visual and parietal cortex and reports of memory uncertainty for spatial location (H.-H. Li et al., 2021) and grating orientation (Geurts et al., 2022). Both of these studies observed a trial-by-trial correlation between a model-based measure of uncertainty of the decoded neural representation and a behavioral report of memory uncertainty, supporting a model whereby observers are directly reading out the quality of their neural representations when making an evaluative judgment of a single WM representation.

These results together are consistent with a model in which all WM representations are random on individual trials due to noise in their neural codes, and participants can read out the quality of an individual cued WM representation and perform comparative judgments across all WM representations. Importantly, our finding that performance is reliably slower on R2-best trials compared with R2-random trials (Fig. 3b) suggests a cost to this comparative judgment, and renders unlikely the possibility that participants report the strongest representation *because* it is the first representation that “comes to mind” during recall. If the strength of the WM representation reliably covaried with RT such that faster responses were associated with stronger representations (e.g., Schneegans & Bays, 2016, 2018), then we would expect our RTs to follow a similar pattern to the response errors shown in Fig. 3a: fastest for R1 trials, slowest for R2-random trials, and intermediate for R2-best trials. This is because, if the two items on R2 trials randomly and independently vary in their strength across trials, then reporting the strongest one (R2-best) should, on average, result in faster responses than randomly reporting either the stronger or weaker one (R2-random). If it were possible to sort R2-random trials based on an estimate of a given trial’s WM representation quality, we would expect that R2-random trials probing the better-remembered item would have faster RTs than those probing the worse-remembered item. Additionally, it could be the case that a color cue at fixation requires an additional level of retrieval (mapping color to position; Schneegans & Bays, 2017) that is unnecessary on R2-best trials, in which the participant in principle could have forgotten color information altogether. Instead, it seems that the longer RTs on R2-best trials compared with R2-random trials index a comparative decision process whereby participants evaluate the quality of each WM item’s representation, compare them, and report the best of the two. Future studies involving whole report tasks (e.g., Adam et al., 2017; Adam & Vogel, 2017) and behavioral confidence reports (e.g., Honig et al., 2020; Li et al., 2021) along with single-trial readouts of the relative quality of multiple WM representations (H.-H. Li et al., 2021; Sprague et al., 2014, 2016) will help better disentangle the cognitive processes supporting these decisions.

### Role of location-based strategies

It has been established that, for features like orientation and color, there exist idiosyncrasies in memory report precision as a function of feature value, such that some colors or orientations can be reported more precisely than others (Bae et al., 2014; Pratte et al., 2017). Perhaps our result is due to our participants adopting a heuristic whereby they choose to report targets from a preferred location or location(s) in the R2-best condition based on their knowledge that they generally can perform better at location recall for items presented at those locations. We pursued this possibility based on some participants reporting to us after the experiment that they seemed to perform better for targets presented (for example) on the left side of the screen compared with the right side. Therefore, we computed the participant's accuracy by location from the R1 condition and compared this with the probability they chose to report an item within that bin during the R2-best condition (Fig. 4a–b). If participants did have better representations in some locations than others, their recall performance should vary substantially for locations at different positions on the screen. Indeed, some participants did show fluctuation in performance at different locations (see example participant data; Fig. 4a), and, in the R2-best condition, some participants did seem to choose some locations to report more than others (Fig. 4b). However, it was rare that the locations chosen were those that could be reported most precisely on R1 trials (cherry-picked example shown in Fig. 4b; see Fig. 4c for all participants). Across our sample of participants, this heuristic could not explain our main result that behavioral performance was improved on R2-best compared with R2-random trials (Fig. 3a). Thus, participants appear to be using information beyond their knowledge of which location(s) on the screen they can remember most accurately when deciding which location to report on R2-best trials, such as the relative quality of their neural code for each item’s location.

### Implications for neural population coding

Modern theories of neural coding posit that information is maintained via activity patterns over populations of neurons. For example, the activity pattern across a population of neurons in primary visual cortex accurately encodes the orientation of an oriented grating which can be decoded using a variety of machine learning methods (Berens et al., 2012; Stringer et al., 2021; Walker et al., 2020). Theoretical (Ma et al., 2006) and empirical (Geurts et al., 2022; Li et al., 2021; van Bergen et al., 2015; Walker et al., 2020) studies have supported the notion that neural population codes additionally (and implicitly) encode the “quality” or “uncertainty” of a representation. Rather than separately keeping track of the feature value represented by a neural population and its uncertainty, both of these types of information are simultaneously encoded in a probability distribution carried by the neural activity pattern. Extensions of this theory to fMRI responses measured in humans have confirmed that participants use these types of uncertainty representations to make judgments about single oriented gratings (Geurts et al., 2022; van Bergen et al., 2015; van Bergen & Jehee, 2019) and spatial positions (H.-H. Li et al., 2021).

The previous behavioral studies testing whether participants can directly compare the quality of multiple WM representations in order to report their best-remembered item (Adam et al., 2017; Fougnie et al., 2012; Suchow et al., 2017; Williams et al., 2022) all allowed participants to compare *feature representations* from different spatial locations. This means that the population code being compared was localized to the retinotopic location for each item (e.g., in Suchow et al., 2017, participants could “look at” the activity profile for neurons with spatial receptive fields corresponding to the screen location of each remembered item, extract the associated uncertainty from these independent populations, and compare these values). However, for spatial locations, such a straightforward processing workflow over disjoint WM representations is not possible. The population that encodes spatial location(s) is necessarily distributed across an entire retinotopic cortical region (and, indeed, may span several of these retinotopic regions across visual, parietal, and frontal cortex; Curtis & Sprague, 2021; Li et al., 2021; Sprague et al., 2014).

For a retinotopic neural population with spatial tuning profiles tiling visual space to simultaneously encode two spatial positions, there must be two “bumps” of activity, each centered at the location of one item. Simple instantaneous readout rules like computing a vector average based on each neuron’s preferred position cannot easily recover each item’s independent position. Instead, the population representation must be “read out” using a strategy that jointly considers the position of multiple objects. This is in direct contrast to scenarios where separate neural populations each encode a feature representation that can be extracted based on their retinotopic locations and compared. Thus, while our result is consistent with previous findings that participants can directly compare the quality of multiple WM representations (Adam et al., 2017; Fougnie et al., 2012; Suchow et al., 2017; Williams et al., 2022), it suggests that this comparison is supported by a more complex type of readout from the population code. Future studies parametrically varying aspects of the task design, including trial-by-trial manipulations of whether location or feature value is reported (e.g., Schneegans & Bays, 2017), may help further clarify how neural codes jointly maintain representations of information and representations of uncertainty for multiple items.

## Data Availability

All experimental data (raw and pre/processed data files) are available on the Open Science Framework (https://osf.io/8c4au/), and analysis code is available on GitHub (https://github.com/SpragueLab/wmChoose_mFiles). Eye-tracking preprocessing and automated scoring procedures are implemented using functions from iEye_ts (github.com/tommysprague/iEye_ts).
